# Role of Histone Deacetylases in Carcinogenesis: Potential Role in Cholangiocarcinoma

**DOI:** 10.3390/cells9030780

**Published:** 2020-03-23

**Authors:** Kishor Pant, Estanislao Peixoto, Seth Richard, Sergio A. Gradilone

**Affiliations:** 1 The Hormel Institute, University of Minnesota, Austin, MN 55912, USA; kpant@umn.edu (K.P.); epeixoto@umn.edu (E.P.); rich1107@umn.edu (S.R.); 2 Masonic Cancer Center, University of Minnesota, Minneapolis, MN 55455, USA

**Keywords:** cholangiocarcinoma, hepatobiliary cancers, HDACs, HDACi

## Abstract

Cholangiocarcinoma (CCA) is a highly invasive and metastatic form of carcinoma with bleak prognosis due to limited therapies, frequent relapse, and chemotherapy resistance. There is an urgent need to identify the molecular regulators of CCA in order to develop novel therapeutics and advance diseases diagnosis. Many cellular proteins including histones may undergo a series of enzyme-mediated post-translational modifications including acetylation, methylation, phosphorylation, sumoylation, and crotonylation. Histone deacetylases (HDACs) play an important role in regulating epigenetic maintenance and modifications of their targets, which in turn exert critical impacts on chromatin structure, gene expression, and stability of proteins. As such, HDACs constitute a group of potential therapeutic targets for CCA. The aim of this review was to summarize the role that HDACs perform in regulating epigenetic changes, tumor development, and their potential as therapeutic targets for CCA.

## 1. Introduction

Cholangiocarcinoma (CCA) is a malignancy arising from cholangiocytes, the epithelial cells of the biliary tree. It is the part of the adenocarcinomas family of tumors also known as bile duct cancer. CCA is a less common malignancy in the liver than hepatocellular carcinoma (HCC), accountable for 10–20% of all liver cancer [1]. CCA is categorized into different groups on the basis of its topography in the liver and bile ducts. If the tumors arise in the liver, it is classified as intrahepatic cholangiocarcinoma (iCCA), and when found outside the liver it is categorized as distal cholangiocarcinoma (dCCA). However, when tumor occurs in the bile duct junctions, it is considered as perihilar cholangiocarcionma (pCCA). Among these, cancer at the perihilar and distal regions are very common, whereas intrahepatic CCA is reported only in 10% of cases [2]. Characteristically, CCA demonstrates non-specific symptoms until a late stage. Inadequate knowledge of the risk factors and correct screening methods make CCA difficult to diagnose at early stages. As a result of frequent late diagnosis, CCA is considered among the deadliest form of cancers, with unfortunate rates of 5-year survival (approximately 5%) in patients [1,3].

**Figure 1:**
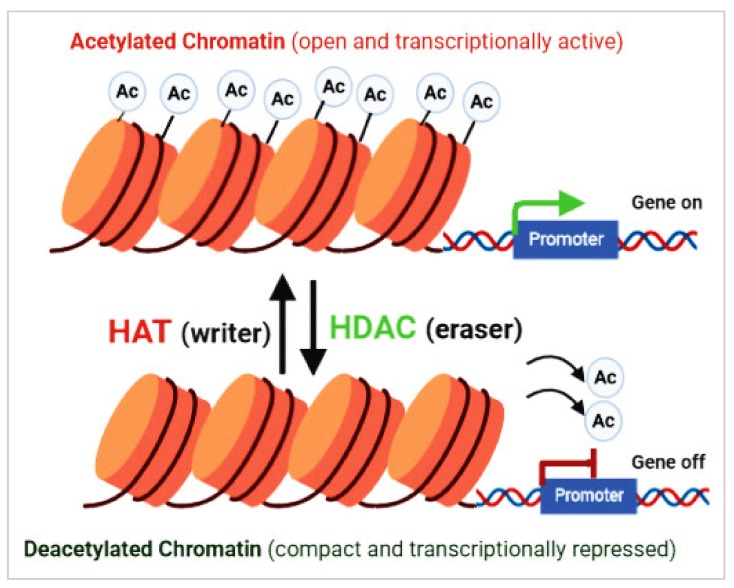
Acetylation and deacetylation of nucleosomal histones and other cellular proteins that play an important role in the modulation of chromatin arrangement and gene expression, as well as in the regulation of protein stability and cellular function. Histone acetyltransferases (HATs) and histone deacetylases (HDACs) are two contrasting classes of enzymes that closely regulate histone acetylation and deacetylation.

Epigenetic modifications, such as acetylation and deacetylation of histones and other cellular proteins, play a vital role in the tumorigenesis and cancer progression. Among these, the importance of histone deacetylase (HDAC)-mediated epigenetic changes in the pathogenesis of CCA has been highlighted [4]. Histone acetylation is a reversible post-translational modification that plays a main role in structure/function of chromatin and in regulating eukaryotic gene expression. Histone/protein acetylation is regulated by the functions of HDACs and histone acetyltransferase (HAT) enzymes [4,5]. HDACs are able to remove acetyl groups from histones and non-histone proteins, and are categorized into two major families, including zinc-dependent and NAD^+^-dependent HDACs (Table 1). HATs catalyze the relocation of an acetyl group from acetyl coenzyme A (acetyl-CoA) to proteins in lysine residues (Figure 1) [4,5]. The equilibrium between histone acetylation and deacetylation is oftentimes deregulated in cancer, causing impaired expression of tumor suppressor genes. HDAC inhibitors (HDACis) are a family of synthetic and natural compounds that differ in their target specificities and activities. HDACis are divided into four main groups on the basis of their structure, including cyclic peptides, benzamides, hydroxamic acids, and short chain fatty acids [4,6,7]. HDACis can significantly affect cancer cells, inducing cell death, cycle growth arrest, angiogenesis reduction, and the immune system modulation [4,7]. This current review mainly focuses on the action of HDACs in the pathogenesis of cancer, including CCA and the therapeutic role of HDACis.

## 2. Classification of Histone Deacetylases

Approximately 18 HDACs have been identified in humans, which have been further classified into four groups on the basis of their homology with yeast HDACs and co-factors. HDAC classes I, II, and IV require a zinc molecule (Zn^+2^) as a cofactor in their active site. As a result, the Zn^2+^ binding HDACis can inhibit these HDACs [8], whereas sirtuins (SIRTs), a class III HDAC, require NAD^+^ as a cofactor rather than Zn^2+^, and are homologous to Sir2 protein in yeast, with similar functions. As a result, Zn^+2^-binding HDACis cannot inhibit class III HDACs (Table 2) [9]. The role of sirtuin proteins in carcinogenesis is dubious, as some SIRT proteins have shown roles as tumor suppressors and/or as oncoproteins [10].

HDACs 1, 2, 3, and 8 are class I HDACs. They are the most abundant class of HDACs present in the nucleus. Class II HDACs can travel between the cytoplasm and nucleus and are larger than the other two Zn^+2^-dependent classes of HDACs, on the basis of domain organization and sequence. HDAC class II is further subdivided into class IIa (HDACs 4, 5, 7, and 9) and class IIb (HDACs 6 and 8), with the latter being characterized as having two deacetylase domains [8]. Class IV has only one HDAC member (HDAC11) [11]. The classification and functions of HDACs are shown in Table 1.

Sirtuins are NAD^+^-dependent class III protein deacetylases, found in bacteria to humans [10,12]. Even though they have diversified functions through evolution, their main functions is to sense changes in redox states of the cell due to intracellular and extracellular stress (i.e., metabolic, oxidative, or genotoxic) and to orchestrate a suitable response [12]. Fluctuations in cellular energy can be sensed by sirtuins by using the NAD^+^ as a cofactor for its enzymatic activity. Even though most of the sirtuins have a wide range of substrates including both histone and nonhistone proteins, some can strictly target to only histones, whereas others have only specificity to nonhistone proteins (Table 1) [12]. Sirtuins include seven members (Sirt1-7) in mammalian cells that may vary extensively in their activity, localization, and functions. Sirt1, 6, and 7 are mainly located in the nucleus, Sirt2 is cytoplasmic, Sirt3 is mitochondrial, whereas Sirt4 and Sirt5 are primarily present in mitochondria [10,13]. Sirtuins can perform numerous functions, including cell survival during stress, metabolic homeostasis, chromatin regulation, and cell differentiation [14,15]. In line with this, sirtuins play an important role at both the cellular and organism levels. They have been associated with diabetes, obesity, cancer, cardiovascular diseases, and also help in viral replication [12,13,16,17], among other disorders.

Histone acetyltransferases (HATs) comprise two main types including A-type (nuclear) and B-type (cytoplasmic). Type-A HATs have various families classified into at least three distinct groups based on structural and functional similarities and, including the *N*-acetyltransferase family, GCN5-related family, family of Moz-Ybf2/Sas3-Sas2-Tip60, and the family of p300/CREB-binding protein (CBP/CREBBP) [18,19]. HAT type-B is highly conserved and has sequence homology with the scHat1 protein [18].

## 3. Histone Deacetylases and Cancer

**Figure 2:**
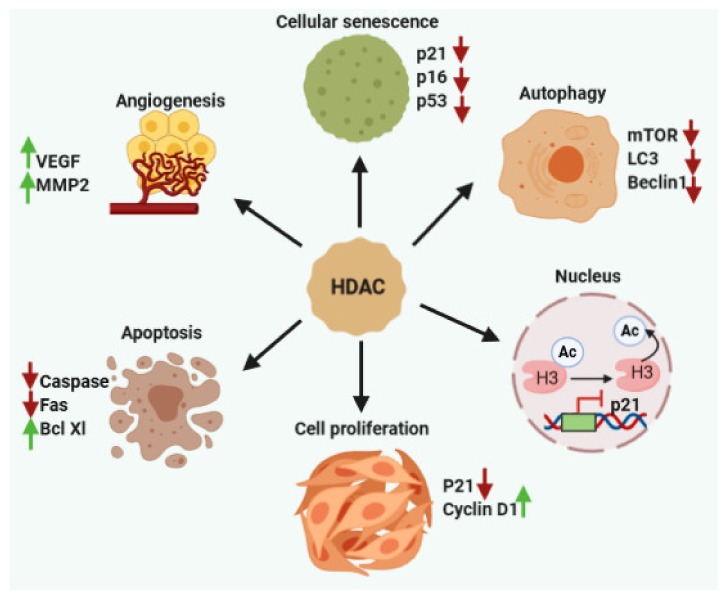
HDACs help in the progression of cancer cell growth and spreading via regulating cell proliferation, angiogenesis, apoptosis, metastasis, autophagy, and senescence.

According to previous studies, dysfunctions of HDAC enzymes and altered level of acetylation are linked to various cancers including CCA [20]. HDACs play a vital role in the epigenetic regulation of gene transcription and expression through their effects on the chromatin compaction state (Figure 1) and affecting the stability of other cellular target proteins [21]. In recent times, HDACs have developed as potential therapeutic targets as these can alter the aberrant epigenetic conditions associated with cancer development. The overexpression of HDACs have been reported in various solid and hematological cancers [22], affecting diverse cellular mechanisms such as proliferation, cell death, metastasis, autophagy, metabolism, and ciliary expression, as described below (Figure 2).

### 3.1. Cell Proliferation

HDACs are known to deacetylate a wide variety of proteins, including the proteins that regulate cell cycle. The cell cycle consists of four phases including G1, S, G2, and M phases. The changes in S phase and M phase are important for genomic integrity [23]. Retinoblastoma protein (pRb) interacts with the E2F members to drive cell cycle progression and apoptosis. pRb can repress E2F-mediated transcription of cell cycle proteins by recruiting HDAC1 to the E2F-responsive promoters [24]. Moreover, HDAC10 has been reported to regulate the cell cycle via modulation of cyclin A2 overexpression, which further rescues G2/M transition arrest in HDAC10 knockdown cells. HDAC10 can regulate cyclin A2 expression by histone deacetylation near let-7 promoter, subsequently repressing transcription [25].

In addition to the transcriptional regulation of cell cycle-linked genes, HDACs also regulate cell cycle progress in transcription-independent manners. HDAC3 is a critical regulator of cell proliferation via a transcription-independent manner, which is recruited by AKAP95 and HA95 along with Aurora B. Deacetylation of histones by HDAC3 induce the Aurora kinase B-mediated maximal phosphorylation of histone H3 on Ser10, leading to progression of cell cycle [26]. In the same way, HDAC3 and HDAC6 induced the cell proliferation and cell survival in CCA cells [27,28].

### 3.2. Cell Death

HDACs have been reported to control apoptosis in various cancer cells via altering expression of apoptotic proteins. Inhibition of HDAC2 leads to the inhibition of tumor cell growth and initiation of apoptosis via activation of p53 and Bax in human lung cancer cells [29]. HDAC3 can down-regulate the expression of PUMA in gastric cancer cells. However, treatment with HDACi promotes PUMA expression through increasing the p53 binding to the PUMA promoter [30]. Aberrant expression of HDAC2 was reported in cancer cells, and treatment with HDAC2 inhibitors reduced cell motility, cell invasion, and cell growth, and induced cell death in gastric cancer cells [31]. In addition, HDAC2 was found to positively regulate Aurora A kinase, which induces pancreatic cancer cell growth and inhibits the cells death via inducing ciliary loss [32].

p53 is a tumor suppressor gene that can induce cell death in the transformed cells. Acetylation of p53 and its role in regulating tumor suppression have been widely explored [33]. When HDACs such as SIRT1 and SIRT2 remove acetyl groups form C-terminal lysines of p53, MDM2 ubiquitinates p53 and leads to its degradation, thus inhibiting p53 level in the cells [34]. Lower levels of p53 in cells may enhance the cell growth and inhibit apoptosis.

### 3.3. Metastasis

Cancer cell invasion and metastasis is an important process in the cancer spreading, and studies have confirmed the important role of HDACs in cancer cell metastasis in a variety of cancers.

Epithelial-cadherin (CDH1) is an epithelial cell marker that is depleted during metastasis. In pancreatic cancer cells, HDAC1 is recruited to the CDH1 promoter, resulting in deacetylation of histone 3 and histone 4 proteins in the nucleus and depletion in E-cadherin, which consequently helps in the epithelial–mesenchymal transition (EMT) [35]. EMT has been recently described as having a prominent role if CCA development; therefore, the regulation of this process by HDACs warrant further investigation [36]. The snail/HDAC1/HDAC2 regulatory axis is essential for EZH2-induced CDH1 inhibition in nasopharyngeal carcinoma cells [37]. ZEB1 regulates the recruitment of HDAC1 and HDAC2 to the CHD1 promoter in human pancreatic cancer cells [38]. SIRT1 also prompts cancer cell migration and metastasis in vivo and in vitro by combining with ZEB1 to inhibit CDH1 expression in prostate cancer cells [39]. Sun et al. also revealed a vital role of SIRT1 and MMP8 interaction in silencing of CDH1 and E-cadherin in prostate cancer cells [40].

### 3.4. Autophagy

The role of autophagy has been extensively studied in the development, maintenance, and progression of cancer cells. Autophagy is not only important for intracellular dynamics, but it has also been observed to control the interaction between the cells and the change in surrounding environment.

Just as autophagy has complex role in cancer development, so too is the effect of autophagy in different members of the Zn^+2^-dependent HDAC enzymes [41]. HDACs such as HDAC6 [28] and HDAC10 induce the deacetylation of cytoplasmic proteins in CCA cells; they have been directly involved in the autophagy process by modulation of key autophagy proteins such as LC3-II and Beclin1 [42]. Cells depleted in class I HDACs were observed with increased autophagic flux, demonstrated by increased autophagosomal proteins such as LC3-II, Beclin1, and ATG5 [43]. On the contrary, HDAC1- and HDAC2-deficient mice had obstructed autophagosome formation [44]. HDAC4 knockdown led to the induction of autophagy, with augmented LC3-II, Beclin-1, and ATG7 levels in the cells [45]. Similarly, HDAC5 downregulation in breast cancer cells led to the increased LC3-II and autophagic flux [46]. According to most studies, depletion of HDACs such as class I and IIa isozymes is linked with higher expression of autophagy regulators involved in the various cell functions. Because autophagy can improve cancer cell viability, simultaneously targeting autophagy might improve the therapeutic effects of HDACi against cancer.

### 3.5. Metabolism

Increased level of HDAC3 was noticed alongside decreased level of pyruvate, subsequently protecting CCA cells from apoptosis. HDAC3 synergistically increased expression of LDHA and PKM2 levels, resulting in low levels of pyruvate, as well as poor survival reports in CCA patients. The authors also found that decreasing levels of pyruvate supported cell proliferation in CCA cells. Elevated HDAC3 activity also stabilized c-MYC protein levels through deacetylation at K323, which further contributed to the low pyruvate levels [47]. In agreement, Yin et al. 2017 also reported that HDAC3 have oncogenic effects in CCA cells by inhibiting apoptosis and contributing to the cell proliferation. In addition, high expression of HDAC3 and HDAC6 was observed in CCA patients’ tissues, linked to low survival [27,28,48].

**Figure 3:**
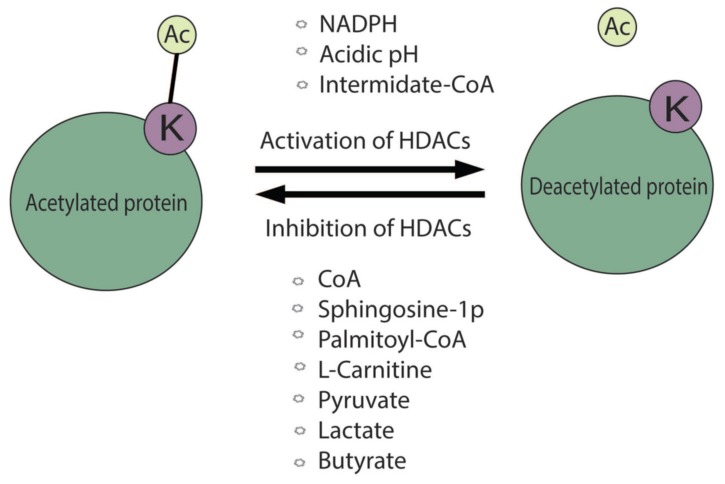
The role of metabolites affecting HDAC activity. In normal conditions, HDACs leads to the removal of the acetyl group from lysine from the target proteins. This reaction is modulated by the described metabolites or physical factors via the activation or inhibition function of HDAC activities.

Several metabolites, produced from various intracellular biochemical reactions, may directly control HDAC activities (Figure 3). The cellular activity of recombinant HDAC1 and HDAC2 complexes were found to be increased with the addition of NADPH and CoA-derivates [49]. In particular, molecules generated from the glycolysis and/or involved in the fatty acid and sterol biosynthesis, including acetyl-CoA, butyryl-CoA, isobutyryl-CoA, succinyl-CoA, malonyl-CoA, methylmalonyl-CoA, and methylcrotonyl-CoA, may increase HDAC activity. In contrast, HDAC1 and HDAC2 activities were inhibited by the long chain fatty acid derivatives such as palmitoyl-CoA and the free CoA [49]. Further studies on the role of metabolic regulation in HDAC activity were observed by a bioactive lipid sphingosine-1 phosphate, formed during nuclear sphingolipid metabolism involved in β-oxidation of fatty acid, which could inhibit HDAC activity by binding to its active site [50,51].

Interestingly, pyruvate and lactate could act as inhibitors of HDACs in different cancer cells. Pyruvate and lactate significantly inhibited HDAC I and HDAC III activities, as comparable to the known HDACIs such as butyrate and trichostatin A (TSA) [52,53].

In a study by McBrian et al., the authors showed that low level of intracellular pH helps in HDAC activity and significantly decreases histone acetylation. Consequently, it releases the free acetate anions in order to prevent further decrease in the pH, suggestive of a relationship between the acidic environment in cancer cells and decreased levels of histone acetylation [54].

Butyrate, a short chain fatty acid that is produced by bacterial fermentation of polysaccharides, can inhibit HDACs [55]. Interestingly, butyrate specially targets cancer cells, whereas it stimulates the growth in normal cells [56]. Further, the researchers validated that the different inhibitory effects in normal and cancer cells is due to differences in the energy metabolism. In non-cancerous cells, the oxidative metabolism is active, which metabolizes butyrate through the tricarboxylic acid (TCA) cycle producing acetyl-CoA that also may favor HAT activity and cell proliferation. On the other hand, cancer cells are characterized as glycolytic in nature, and therefore uses a moderately small amount of butyrate through the TCA cycle, enabling butyrate to accumulate in the cell, acting as an HDAC inhibitor [56]. In addition, β-hydroxybutyrate (βOHB), another source of energy for cells, produced during prolonged physical activities and starvation, has also been found to be an inhibitor of HDAC (HDAC1, HDAC3, and HDAC4) activities [57].

### 3.6. Ciliary Assembly

The primary cilium is a sensory organelle expressed in almost every cell of the body, including cholangiocytes [58,59]. Several studies have reported that primary cilia can serve as antennae, which have mechanical and chemical sensory capabilities to communicate with the extracellular environment. As the primary cilia act as sensory organelles of the cells, deficiencies in primary cilia formation may lead to various disorders in humans, or ciliopathies. Disorders linked with genetic mutations in ciliary proteins often lead to abnormality in cilia formation. Ciliary dysfunction has been associated with intellectual disabilities, Bardet–Biedl syndrome, polycystic kidney disease, obesity, oral facial syndrome, cancer, and others [58,60,61]. Specifically, decreased or distorted primary cilia in cholangiocytes have important clinical implications and are associated with many biliary diseases, including CCA [62,63].

**Figure 4:**
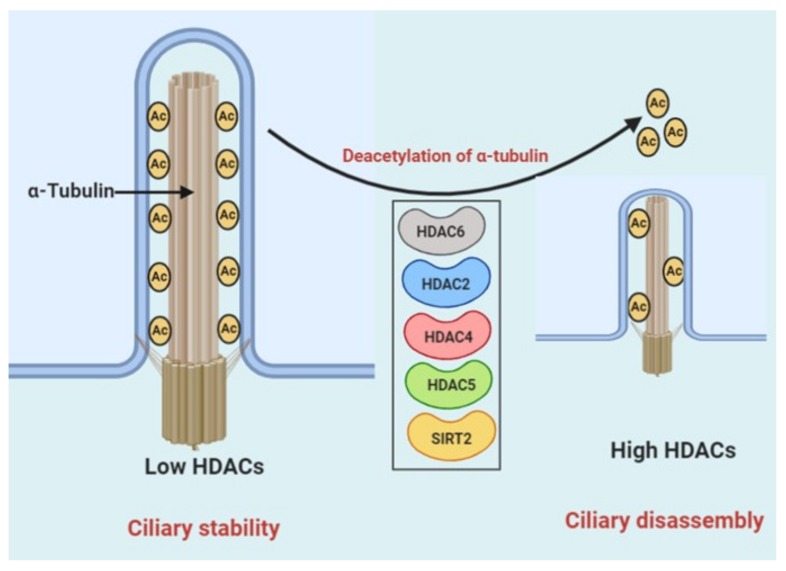
Working model for the role of HDACs in ciliary disassembly. Ciliary disassembly is mediated through deacetylation of α-tubulin in the ciliary axoneme mediated by HDACs. Removal of acetyl groups from α-tubulin destabilizes axoneme microtubules, promoting ciliary resorption.

HDAC6 is known to inhibit ciliary assembly in CCA cells via deacetylating the tubulin protein in the ciliary axoneme [48,64]. Previously, we reported that HDAC6 expression was noticed as being upregulated in CCA cells [28] due to a dysregulation of miRNA-433 and miRNA-22, which induces the expression of HDAC6, consequently prompting ciliary loss and cell growth in normal cholangiocytes [65]. Interestingly, exposure to cigarette smoke induced HDAC6-mediated deacetylase activity, which led to autophagy-mediated cilia shortening in epithelial cells of mouse models [66]. Additionally, in pancreatic ductal adenocarcinoma cells, HDAC2 has been reported as a regulator of primary cilium formation, which reduces the expression of Aurora A kinase and thereby stimulates disassembly of primary cilia [32]. In zebrafish, HDAC4 and HDAC5 modulate cilia formation during kidney morphogenesis [4]. In another study in zebrafish, decreased cilia numbers and uncharacteristic centrosome amplification and polyploidy were observed with overexpression of SIRT2 in Kupffer’s vesicle. Reduction of SIRT2 inhibited the irregular amplification of centrosome and polyploidy associated with loss of polycystin-1 (PC1) [67] (Figure 4).

#### HDACs Inhibitors (HDACis) and CCA

HDACis can inhibit the HDAC activities, increase the aggregation of histone acetylation in autosomes, and stimulate gene expression [4]. Many previous studies have shown that HDACis can exert anticancer effects through several mechanisms inducing cell cycle arrest, induction of apoptosis, and autophagy in cancer cells [66,67].

The FDA has approved a number of HDACis such as vorinostat and romidepsin, which can target chromatin remodeling. These inhibitors have increased therapeutic efficacy in hepatobiliary cancer including CCA. HDACi (MS-275) treatment effectively inhibits the cell growth in EGI-1 and TFK-1 CCA cells by inducing cell cycle arrest and apoptosis [22]. Apoptosis was accompanied by the activation of caspase-3, Bax, and downregulation of Bcl-2. Cell cycle arrest is mostly inhibited at the G1/S checkpoint, which is associated with the induction of p21Waf/CIP1, an inhibitor of cyclin-dependent kinase [44,45]. MI192 has been shown to inhibit the deacetylase activity of HDAC3 in CCA, leading to reduced cancer cell growth in vitro and vivo [27]. HDAC3 was noticed as being upregulated in CCA cells as compared to adjacent normal cells. Taken together, HDAC3 was inhibited by MI192 and prompted apoptosis in human CCA cells [27].

Furthermore, application of cisplatin is related with drug resistance, implying the need for urgent development of improved combination therapies. In clinical application of HDAC inhibitors, including suberoylanilide hydroxamic acid (SAHA) and trichostatin A (TSA), synergistically induced anticancer effects occurred in CCA cell growth with cisplatin. Cisplatin, when combined with TSA or SAHA, synergistically led to cell growth inhibition and apoptosis in CCA cell lines such as KKU-M214 and KKU-100 [68]. The expression of HDAC1 and 2 was upregulated in the CCA cell lines. In other studies, the combination of 5-flourouracil and HDACis such as SAHA and TSA inhibited the cell growth in CCA cells [69] and administration of HDACi CG200745, along with other chemotherapeutic agents including cisplatin, 5-fluorouracil, and oxaliplatin, and decreased CCA cell viability via modulating the Hippo signaling pathway by inducing expression of miR-509-3p [70].

HDAC6 is another isoform of HDACs that is considered as enhancing CCA cell growth [58].

Inhibitors of HDAC6 such as ACY1215 and tubastatin-A significantly inhibited CCA cell growth in vitro and in vivo [28,71,72].

In gallbladder cancer, SAHA treatment inhibits cell proliferation by activating tumor suppressor gene p21 in the cells. Treatment of SAHA also caused significant decline in cell number in carcinoma cells when used synergistically with EZH2 siRNA; conversely, the expression of EZH2 and HDAC1/2 were decreased by SAHA treatment in TGBC2TKB cells [73]. The combination of SAHA and TSA reduces the cell growth and induces apoptosis in gallbladder carcinoma SGC-996 cells. Furthermore, they downregulate the phosphorylation of Akt and mTOR and inhibit the expressions of c-Myc, cyclin D1, and Bmi1 [74]. Some experimental studies have demonstrated that HDACi PCI-24781 have an anticancer effect on human gallbladder carcinoma cell and in mice. This effect is associated with downregulation of erbB2 protein activity and increased levels of tubulin and histone acetylation. PCI-24781 also resulted in effective inhibition of gemcitabine-resistant gallbladder carcinoma cell growth [75].

Drugs, including clofibratem, imexon, gefitinib, ciprofloxacin, and dexamethasone, have the capability to induce the formation of primary cilia in cancer cells [76]. These drugs also inhibit cell growth and may induce apoptosis. Because overexpression of HDAC6 in cholangiocytes induces ciliary loss; HDAC6 is a novel therapeutic target for ciliopathy-related disorders. HDAC6-mediated deacetylation of α-tubulin disrupts the stability of the axoneme leading to ciliary loss [7,10]. A previous study demonstrated that high expression of HDAC6 induced loss of primary cilia in CCA and polycystic liver disease (PLD) [28,72]. Consequently, the application of HDAC6 inhibitors to block the malignant phenotype in CCA cells could be a promising therapeutic strategy. CCA growth was significantly inhibited in in vitro and in vivo models by HDAC6 inhibitors via inducing re-expression of cilia [28]. Thus, the restoration of the primary cilia may be a potential therapeutic approach for CCA, and HDAC6 inhibitors may be important agents for the treatment of other ciliopathy-related diseases [77].

## 4. Natural Compounds Targeting HDACs

Natural compounds provide potent and pleiotropic ranges of drugs. Various HDACis discovered thus far are of natural origin. HDACi such as FK322, a cyclic peptide isolated from *Chromobacterium violaceum*, as well as TSA from *Streptomyces hygroscopicus*, selectively prevent HDAC1 and 2 activities. Trapoxin A and depudecin are also naturally occurring HDACis extracted from a fungus. Some marine organisms are also the source of potent natural HDACis, such as largazole and azumamides [6,78].

Other natural HDAC inhibitors such as KK4 and ICG15042, isolated from peanut skin extract, inhibited the growth of CCA cell via apoptosis induction and cell proliferation inhibition [79].

Epigallocatechin-3-gallate (EGCG) is a potent cancer chemopreventive and therapeutic agent isolated from the tea polyphenols, and is the major green tea polyphenol extract [80,81]. EGCG has been reported to have anticancer properties via inhibition of the HDAC activities in various cancers including lung, cervical, and melanoma [79,80,81,82,83,84].

Butyrate is a short chain fatty acid produced by the gut microbiota during fermentation of the dietary fibers, and has been shown to affect post-translational modifications of histones by inhibiting HDAC class III [85,86]. Butyrate has been reported to inhibit growth in hepatocellular carcinoma [14,87,88], lung cancer [89], breast cancer [90], pancreatic cancer [91,92], and colon cancer cells via increasing the acetylation of histones [56,93]. However, the role of butyrate in CCA has not been studied thus far.

## 5. Conclusions

Previous studies in the last decade have validated the fact that HDACs can play a critical role in cancer development by controlling acetylation of histone and non-histone proteins. As an eraser of acetylation from proteins, HDACs have been found to enhance oncogenic functions, suggestive of a crucial attractive therapeutic target against CCA. HDACs may play a main role in carcinogenesis through many pathways, and HDACis can prevent HDAC activity and work as an anticancer strategy by affecting multiple mechanisms inducing cell growth inhibition, cell cycle arrest, apoptosis, and restoration of cilia formation in CCA cells. These compounds can enhance histone acetylation levels, by which they control the expressions of genes that are involved in various biological pathways in cancer cells. Together, a good understanding of the HDAC- and HDACi-mediated molecular mechanism and pathway could lead to a new window toward developing a new therapeutic target against CCA.

## Figures and Tables

**Table 1:** Different classes of HDACs, their co-factors, and their cellular locations.

Co-Factor	Class	Members	Location
Zn^+2^-dependent	HDAC I	HDAC1	Nucleus
		HDAC2	Nucleus
		HDAC3	Nucleus
		HDAC8	Nucleus
	HDAC II	HDAC4	Nucleus/cytoplasm
		HDAC5	Nucleus/cytoplasm
		HDAC7	Nucleus/cytoplasm
		HDAC9	Nucleus/cytoplasm
		HDAC6	Cytoplasm
		HDAC10	Cytoplasm
	HDAC IV	HDAC11	Nucleus
NAD^+^-dependent	HDAC III	SIRT1	Nucleus/cytoplasm
		SIRT2	Nucleus
		SIRT3	Mitochondria
		SIRT4	Mitochondria
		SIRT5	Mitochondria
		SIRT6	Nucleus
		SIRT7	Nucleus

**Table 2:** Targets of different HDAC inhibitors (HDACis). **SAHA**—suberoylanilide hydroxamic acid, **TSA**—trichostatin A, **SCFA**—short chain fatty acids.

HDAC Inhibitors	Activity	Target HDACs
**SAHA**	Hydroxamates	I, II, IV
**TSA**	Hydroxamates	I, II, IV
**Ky2**	Hydroxamates	I
**Apicidine**	Depsipeptides	I
**FK228**	Depsipeptides	I
**VPA**	SCFA	I, II
**Butyrate**	SCFA	I, IIa
**4-BP**	SCFA	I, IIa
**MS-275**	Benzamides	I
**Cl-994**	Benzamides	I
**MGCD0103**	Benzamides	I, IV
**LAQ842**	Other	I, II, IV
**MY192**	Other	I
**PXD101**	Other	I, II, IV
**LBX589**	Other	I, II, IV
**MPT0G009**	Other	I, II, IV
**ACY1215**	Other	II
